# Electrophysiological Anomalies in Face–Name Memory Encoding in Young Binge Drinkers

**DOI:** 10.3389/fpsyt.2017.00216

**Published:** 2017-11-01

**Authors:** Rocío Folgueira-Ares, Fernando Cadaveira, Socorro Rodríguez Holguín, Eduardo López-Caneda, Alberto Crego, Paula Pazo-Álvarez

**Affiliations:** ^1^Department of Clinical Psychology and Psychobiology, University of Santiago de Compostela, Galicia, Spain; ^2^Neuropsychophysiology Laboratory, CIPsi, School of Psychology, University of Minho, Braga, Portugal

**Keywords:** memory encoding, difference memory effect, face–name association, binge drinking, college students

## Abstract

A growing body of evidence indicates that the intake of large amounts of alcohol during one session may have structural and functional effects on the still-maturing brains of young people. These effects are particularly pronounced in prefrontal and hippocampal regions, which appear to be especially sensitive to the neurotoxic effects of alcohol. However, to date, few studies have used the event-related potentials (ERPs) technique to analyze the relationship between binge drinking (BD) and associative memory. The objective of this study was to examine brain activity during memory encoding using the *Subsequent memory paradigm* in subjects who have followed a BD pattern of alcohol consumption for at least 2 years. A total of 50 undergraduate students (mean age = 20.6 years), i.e., 25 controls (12 females) and 25 binge drinkers (BDs; 11 females), with no personal or family history of alcoholism or psychopathological disorders, performed a visual face–name association memory task. The task used enables assessment of the *Difference due to memory effect* (Dm), a measure of memory encoding based on comparison of the neural activity associated with subsequent successful and unsuccessful retrieval. In ERP studies, study items that are subsequently remembered elicit larger positive amplitudes at midline parieto-frontal sites than those items that are subsequently forgotten. The Dm effect generally appears in the latency range of about 300–800 ms. The results showed a Dm effect in posterior regions in the 350–650 ms latency range in the Control group. However, in the BD group, no significant differences were observed in the electrophysiological brain activity between remembered and forgotten items during the encoding process. No differences between groups were found in behavioral performance. These findings show that young BDs display abnormal pattern of ERP brain activity during the encoding phase of a visual face–name association task, possibly suggesting a different neural signature of successful memory encoding.

## Introduction

Binge drinking (BD) is a pattern of alcohol consumption characterized by the intake of five or more drinks (four or more for females) on a single occasion within a 2-h interval, reaching blood alcohol concentration to 0.08 g/dL (1) at least once in the last month (2).

The most recent report of the World Health Organization (3) has highlighted that the highest rates of BD among young people occur in Europe (31.2%), the USA (18.4%) and the Western Pacific Region (12.5%). Furthermore, the rate reaches 41.8% in the 18–25 age range (4). BD has become a major concern for public authorities because of its adverse impact on a wide range of personal, social, and health issues and also because of the associated economic cost.

Adolescence is a critical stage of development in which the brain undergoes processes of neuromaturation and reorganization (5), which extend into the third decade of life. In accordance with animal studies, this period is particularly sensitive to the effects of BD, which causes more brain damage in adolescent than in adult rats, especially in the prefrontal cortex and the hippocampus (6, 7). It has also been shown that these alterations can lead to long-lasting changes in the adult brain (8, 9).

To date, most of the relevant research in humans has focused on the consequences of this pattern of alcohol consumption on the still-maturing brain (10). Neuropsychological studies have shown that BDs display greater difficulties in processes such as working memory (11), inhibitory control (12), decision-making (13), or declarative memory (14). Functional magnetic resonance imaging (fMRI) studies have also reported abnormal brain activity in BDs during verbal learning tasks (15, 16), affective decision-making (17), working memory (18, 19), or inhibitory control (20). Furthermore, event-related potential (ERP) studies have demonstrated anomalies in BDs in different components related to processes such as attention (21), working memory (22, 23), inhibitory control (24, 25), emotional auditory processing (26), or reactivity to alcohol-related cues (27, 28).

Despite the growing evidence from research on the neurocognitive consequences of BD, few studies have examined brain activity related to associative memory processes in BDs. One type of associative memory, which has a key role in the social context, is the association between names and faces. Neuroimaging studies have shown that the encoding of face–name associations in intramodal (29–31) and intermodal tasks (32, 33) involves a network of brain structures, including the fusiform gyrus, the hippocampal formation and the dorsolateral prefrontal cortex. Scientific evidence regarding BD has reported alterations in regions such as the hippocampus and the prefrontal cortex, and it is, therefore, possible that associative memory may be impaired in BDs.

The *Subsequent memory paradigm*, in which the neural activity is recorded while individuals are explicitly or implicitly memorizing specific items, is a particularly powerful approach to studying memory encoding. The stimuli are classified on the basis of whether they were remembered or not in a subsequent memory test. In general, fMRI studies have revealed that medial temporal structures and prefrontal regions show greater activity for remembered than for non-remembered items, and this increased activity is assumed to reflect successful encoding processes. This effect, referred to as *difference due to memory effect* (Dm) or differential neural activity based on memory (34, 35), has been observed for a variety of stimuli, including faces, words, and objects (34).

Event-related potential approaches to studying declarative memory during encoding have also demonstrated the Dm effect. The ERPs elicited by study items that are subsequently remembered show larger positive amplitudes than ERPs elicited by subsequently forgotten items in midline parieto-frontal regions (36, 37). The Dm effect generally appears in the latency range of about 300–800 ms or even later, and it has been shown to be modulated by the type of encoding material (verbal vs non-verbal), task instructions (incidental vs intentional), and the type of encoding (deep vs shallow; associative vs non-associative) (35, 38). Furthermore, the effect is stronger when memory formation is intentional, associative, and requires free-recall judgments (35, 39).

In the present study, we recorded the ERPs elicited while participants performed a face–name pairs association task with subsequent memory testing, in order to shed further light on potential memory deficits associated with BD consumption in university students. We hypothesized that BD would impair face–name memory encoding at an electrophysiological and/or behavioral level because of the role of the hippocampus and the prefrontal regions in associative memory encoding, and taking into account previous reports of the influence of alcohol intake on these regions. The associative task used in this study is characterized by intentional encoding and cued-recall judgments and is, therefore, expected to elicit a clear Dm effect.

## Materials and Methods

### Participants

The sample comprised 50 undergraduate students. The participants were selected from among first-year students at the University of Santiago de Compostela (Spain) who voluntarily filled in a questionnaire administered in class. The questionnaire included the Alcohol Use Disorders Identification Test (40) and other questions about substance use [for further details, see Ref. (41)]. From this initial screening, 164 subjects were enrolled in a longitudinal neurocognitive study, after undergoing a semi-structured interview in which more detailed inclusion and exclusion criteria were verified. Those participant who (1) drank six or more standard alcoholic drinks on the same occasion, one or more times per month, or (2) during these episodes, drank at a speed of consumption of at least three drinks per hour, were classified as BDs. Those who (1) drank six standard alcoholic drinks on the same occasion less than once per month and (2) drank at a maximum speed of consumption of two drinks per hour were classified as Controls. Exclusion criteria comprised non-corrected sensory deficits, any episode of loss of consciousness for more than 20 min, history of traumatic brain injury or neurological disorder, personal or familial (first-degree) history of psychopathological disorders (according to DSM-IV criteria), use of illegal drugs except cannabis, and scores above 20 in the Alcohol Use Disorders Identification Test. Two years (mean 22 months) after the first neurocognitive evaluation, participants were called for a follow-up. They were interviewed again, and those who continued to fulfill the inclusion and exclusion criteria completed a new neurocognitive assessment in which they carried out the face–name task reported here (as well as other tests). Twenty-five (11 females) subjects from the BD group and 25 (12 females) from the Control group participated in the present study (mean age 20.6 ± 0.66 years). The main demographic data and alcohol and drug use habits of the participants in the follow-up study are summarized in Table 1.

**Table 1:** Demographic and drinking characteristics of the Control and BD groups.

	Control	Binge drinkers	*p*-Value
Gender (M/F)	13/12	14/11	ns
Age	20.48 ± 0.58	20.76 ± 0.72	ns
Handedness (right/left)	23/2	22/3	ns
Caucasian ethnicity (%)	100	100	ns
Regular tobacco smokers	0	9	0.001*
Regular use of cannabis^a^	0	4	0.070
SCL-90-R: GSI percentile scores	37.20 ± 29.93	38.40 ± 31.61	0.891
Age of onset of drinking	15.52 ± 1.03	14.56 ± 1.36	0.011*
Total grams of alcohol in a standard week	39.3 ± 35.92	251.6 ± 136.66	0.001*
Speed of consumption: drinks per hour	0.90 ± 0.76	2.56 ± 0.82	0.001*
Number of drinks in a standard drinking episode	1.98 ± 1.28	5.62 ± 1.79	0.001*
Percentage of times became drunk when drinking	14.28 ± 21.19	58.40 ± 26.25	0.001*
Total AUDIT score	2.80 ± 2.35	11.68 ± 3.62	0.001*

*^a^Once or more a week*.

*p < 0.05.

*ns, non-significant; SCL-90-R, Symptom Checklist-90-Revised; AUDIT, Alcohol Use Disorders Identification Test*.

Informed consent was obtained from all subjects, who were paid for their voluntary participation in the study. The experiment study was undertaken in compliance with Spanish legislation and the Code of Ethical Principles for Medical Research Involving Humans Subjects outlined in the Declaration of Helsinki.

### Procedure

Subjects were asked to abstain from consuming alcohol and other drugs for 24 h before the experiment to prevent effects of acute alcohol intake and to rule out withdrawal effects. In addition, they were instructed not to smoke or drink tea or coffee for at least 3 h before the assessments.

Participants were seated in an electrically shielded, sound attenuated, and dimly lit room at a viewing distance of 100 cm from a 21″ video CRT monitor (1,024 × 768 at 60 Hz). Each stimulus consisted of a face (2.9°× 3.4° visual angle) projected on a gray background and with a fictional first name printed underneath. The pictures, depicting 108 unfamiliar faces (half were female), were extracted from the AR Face Database (42). The images were cropped, resized, oval masked, and converted to gray level images using ImageMagick. All faces had a neutral expression.

During the study phase of the experiment, participants were asked to form associations between each face and the corresponding name and to try to memorize them. Face–name pairs were arranged in 18 encoding blocks (six pairs per block) presented for 1.5 s and followed by a 2–3 s randomly varying inter-stimuli interval.

**Figure 1:**
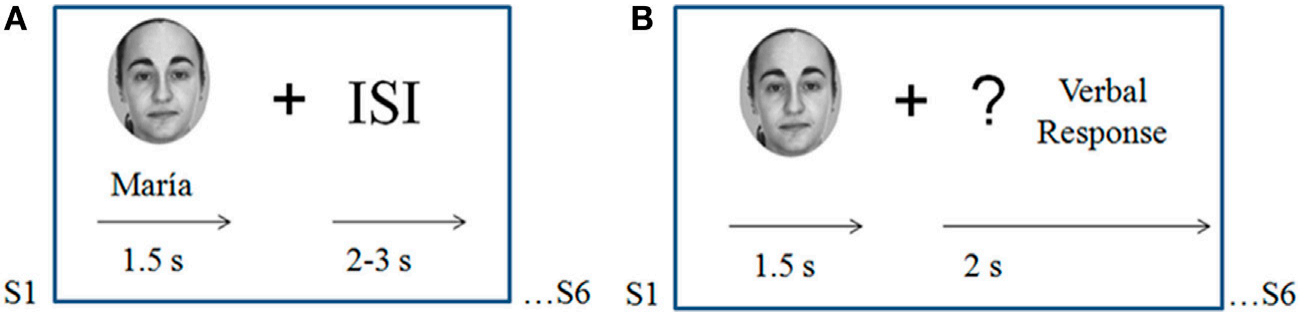
Task design. **(A)** Study phase (encoding blocks): participants were asked to memorize novel face–name associations. Pairs were presented for 1.5 s each with a variable inter-stimulus interval of 2–3 s. **(B)** Recall blocks: the six faces memorized during the precedent encoding block were presented alone for 1.5 s; participants were instructed to verbally recall the name associated with each face when a question mark appeared on the screen.

Immediately after each encoding block, participants were presented with a recall block that consisted of a cued-recall test for names in which each of the six memorized face stimuli were presented (in a different order than in the learning block) for 1.5 s, followed by a question mark that remained in the center of the screen for 2 s. Participants were instructed to verbally recall the name that matched the face presented. Responses were allowed only after the face had disappeared from the screen, during presentation of the question mark (Figure 1).

Faces and names were never repeated during the encoding blocks of the experiment to ensure that the brain activation during this phase only reflected encoding processes and not automatic retrieval (recognition of familiar faces).

### Electroencephalogram (EEG) Recording and Processing

The electroencephalogram was recorded with Brain Vision amplifiers (BrainAmp), using an Easycap with 32 synterized Ag–AgCl electrodes placed at AF3, AFz, AF4, F7, F3, Fz, F4, F8, FC3, FCz, FC4, C3, Cz, C4, CP3, CPz, CP4, T7, T8, P7, P3, Pz, P4, P8, PO7, PO3, POz, PO4, PO8, O1, Oz, and O2 (according to the extended 10–20 International System). All active electrodes were referred to the nose tip and grounded with an electrode placed at Fpz. Vertical electrooculogram was recorded bipolarly from above and below the left eye to control eye movements and blinks. Electrode impedances were kept below 20 kΩ. EEG signals were continuously amplified and digitized at a rate of 500 Hz, and filtered on-line with a 0.01–100 Hz band pass filter.

Electroencephalogram data were off-line processed with Brain Vision Analyzer software (Version 2.0). Ocular artifacts were corrected by the procedure developed by Gratton et al. (43). The data were then digitally filtered with a 0.1–30 Hz bandpass filter (24 dB/oct) and segmented into epochs of 1000 ms, from 100 ms pre-stimulus to 900 ms post-stimulus. Those which exceeded ±90 μV were rejected and baseline-corrected.

Epochs recorded during encoding blocks were averaged separately according to the participant’s memory judgments in the subsequent cued-recall test. Therefore, two conditions per group were computed: correctly encoded (CE) face–name pairs and incorrectly encoded (IE) pairs. There were no statistical differences in the number of averaged epochs between the groups for the CE (Controls: 51.04 ± 16.69; BD: 44.76 ± 14.36) or IE condition (Controls: 36.16 ± 15.49; BD: 39.84 ± 15.46).

Several measures were extracted for each averaged ERPs: (1) the Dm, an index of memory encoding that was the focus of this study, was quantified by comparing the mean ERP amplitudes elicited during encoding of pairs that were later remembered or forgotten in three separate latency intervals (200–350, 350–500, and 500–650 ms) at centroparietal (mean amplitude of the sites CP3, CPz, CP4, P3, Pz, and P4 at each latency interval), and parieto-occipital (mean amplitude of sites PO3, POz, PO4, O1, Oz, and O2) regions. These latency windows were selected to cover the deflection where Dm is apparent by visual inspection of the grand-averages and adjusted so that they comprised 150 ms equal length and covered all positive deflection. (2) N170 and vertex positive potential (VPP) were measured to confirm whether the task elicited the usual ERP pattern for perceptual processing of faces. N170 was identified as the mean amplitude in the 140–180 ms post-stimulus interval at P7, PO7, P8, and PO8 electrode sites; VPP was measured as the mean amplitude in the same latency interval at C3, Cz, and C4.

### Data Analysis

Preliminary analysis considering Gender and Laterality (left vs right hemisphere) did not indicate any significant main effects and/or interactions of these factors, and therefore they were not included in subsequent analyses.

Behavioral performance was analyzed by using a Student’s *t*-test to compare (Control vs BD) the percentage of hits (subsequently recalled names associated with faces).

Regarding the ERPs, analysis of variance (ANOVA) was conducted for the Dm effect in a 2 × 2 × 2 mixed-model design, with two within-subject factors, Condition (CE vs IE) and Region (Centroparietal vs Parieto-occipital), and one between-subject factor, Group (Control vs BD) for each of the three measured latency intervals. N170 was analyzed using a 2 × 4 × 2 mixed-model design with Condition (CE vs IE) and Electrode (P7, PO7, P8, and PO8) as within-subject factors, and Group (Control vs BD) as a between-subject factor, and VPP in a 2 × 3 × 2 mixed-model design with Condition (CE vs IE) and Electrode (C3, Cz, C4) as within-subjects factors, and Group (Control vs BD) as a between-subject factor.

Alpha levels were considered at 0.05 and the degrees of freedom were corrected, when appropriate, by the Greenhouse-Geisser estimate. *Post hoc* paired comparisons were performed with the Bonferroni adjustment for multiple comparisons.

## Results

### Behavioral Performance

The percentage of correctly recalled names (Control = 58.59 ± 13.2%; BD = 55.55 ± 13.3%) was equivalent in the two groups [*t*(48) = 0.808, *p* = 0.423].

### Event-Related Potentials

**Figure 2:**
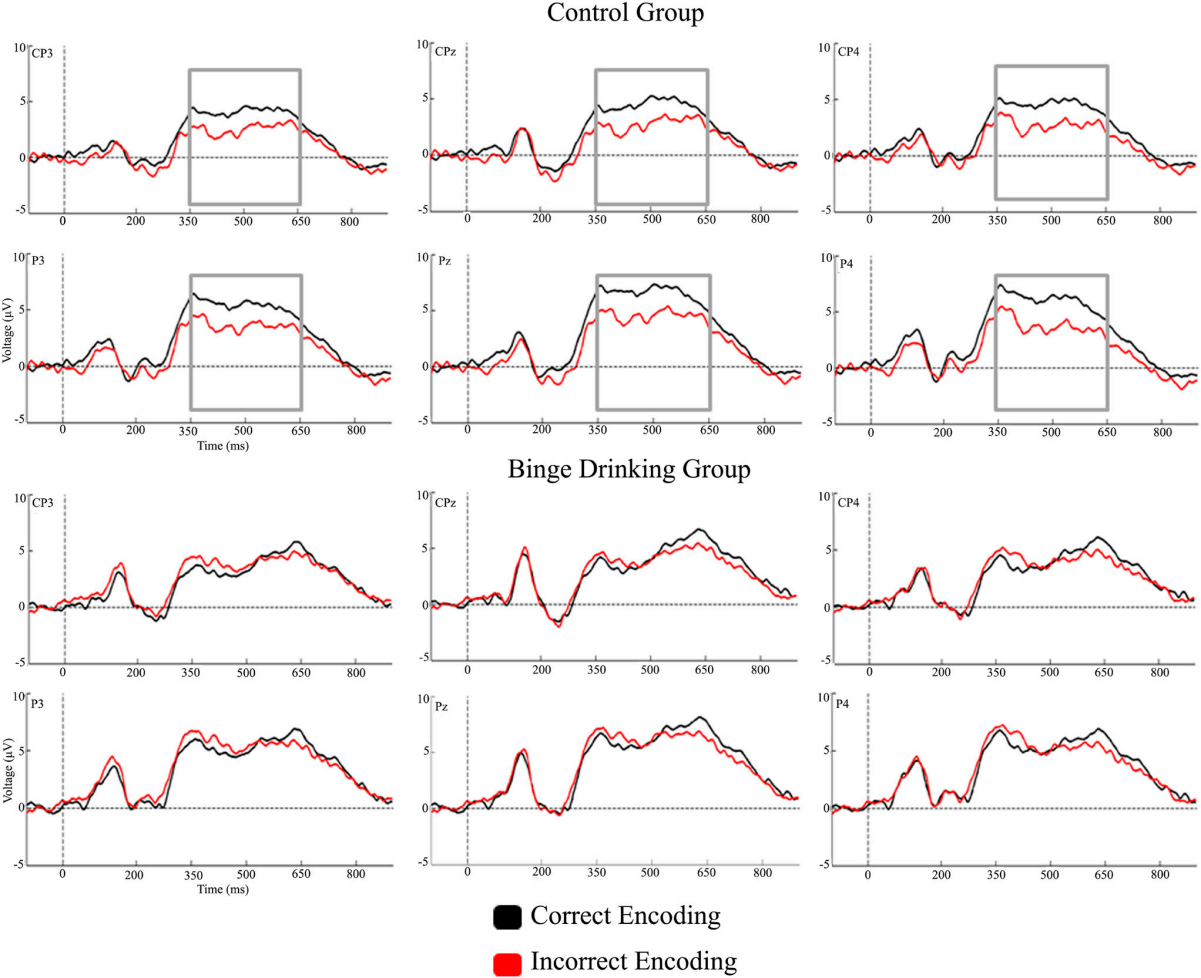
Grand-averages of event-related potentials from Control and binge drinking groups at centroparietal region.

**Figure 3:**
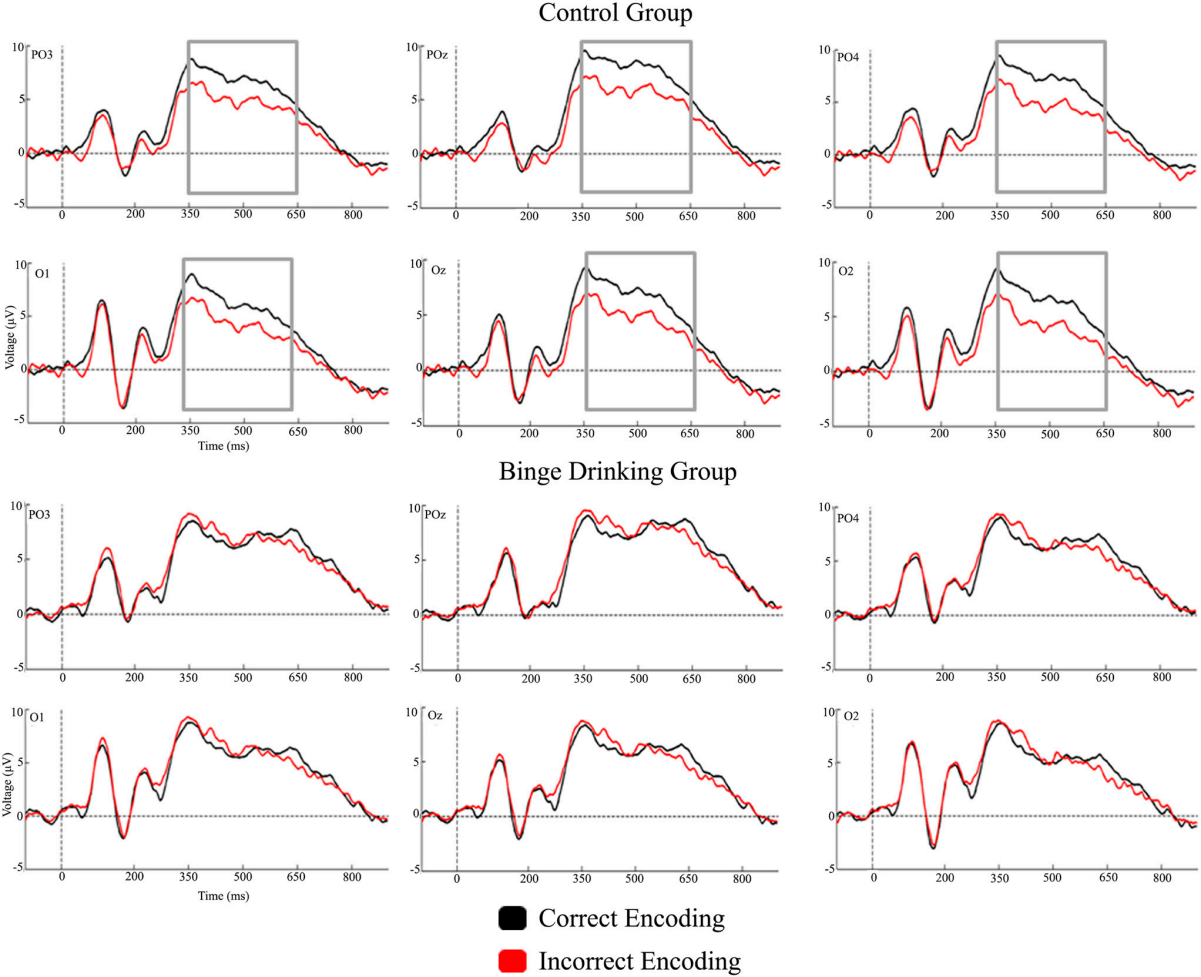
Grand-averages of event-related potentials from Control and binge drinking groups at parieto-occipital region.

**Figure 4:**
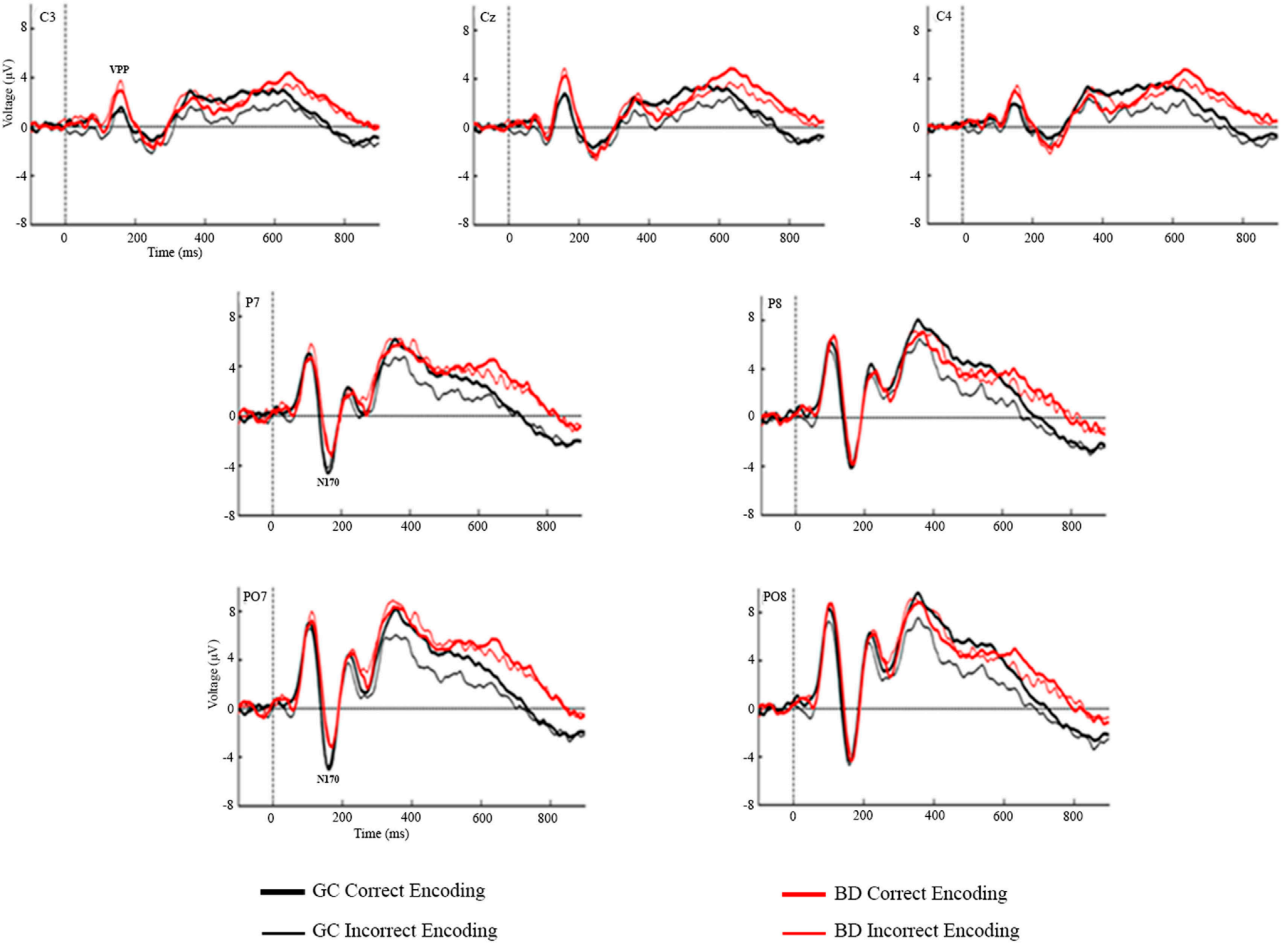
Grand-averages of event-related potentials from Control and binge drinking groups at C3, Cz, C4 (VPP) and at P7, P8, PO7, and PO8 electrodes (N170).

Figures 2 and 3 represent the grand-averages of the ERP at the centroparietal and parieto-occipital sites considered to evaluate the Dm. Figure 4 represents the grand-averages corresponding to the electrode sites considered to evaluate N170 and VPP.

Table 2 presents the descriptive statistics of the amplitude values for the two groups and conditions at the three latency windows used to analyze the Dm.

**Table 2:** Mean amplitude (μV) in the CE and IE conditions (mean ± SD) at the 12 electrodes analyzed in Control and BD groups at 200–350, 350–500, and 500–650 ms latency intervals.

	BD						CG					
	200–350 ms		350–500 ms		500–650 ms		200–350 ms		350–500 ms		500–650 ms	
	CE	IE	CE	IE	CE	IE	CE	IE	CE	IE	CE	IE
CP3	0.77 ± 2.94	1.31 ± 2.33	3.34 ± 3.61	3.92 ± 4.32	4.94 ± 3.48	4.46 ± 4.79	0.72 ± 3.27	−0.10 ± 4.23	4.00 ± 4.28	2.36 ± 5.34	4.29 ± 4.26	2.95 ± 4.72
CPz	0.58 ± 2.95	0.71 ± 2.48	3.68 ± 3.72	3.92 ± 4.22	5.83 ± 3.22	4.94 ± 4.57	0.40 ± 3.41	−0.41 ± 4.35	4.29 ± 4.68	2.52 ± 5.60	4.71 ± 4.66	3.25 ± 4.92
CP4	1.11 ± 2.72	1.35 ± 2.57	3.82 ± 3.58	4.27 ± 4.24	5.26 ± 3.03	4.48 ± 4.22	1.23 ± 3.33	0.60 ± 4.38	4.62 ± 4.53	2.90 ± 5.73	4.58 ± 4.33	2.84 ± 4.64
P3	2.25 ± 2.90	2.79 ± 2.76	5.31 ± 3.84	5.81 ± 4.92	6.24 ± 3.45	5.70 ± 5.11	1.81 ± 3.41	0.82 ± 4.66	5.67 ± 4.42	3.79 ± 5.49	5.22 ± 4.68	3.66 ± 5.10
Pz	1.97 ± 3.22	2.29 ± 2.76	6.02 ± 4.01	6.38 ± 4.84	7.49 ± 3.23	6.70 ± 4.85	1.78 ± 3.52	0.59 ± 4.75	6.77 ± 4.62	4.59 ± 5.76	6.49 ± 4.93	4.74 ± 5.12
P4	2.77 ± 2.87	3.05 ± 2.87	5.62 ± 3.80	6.01 ± 4.69	6.24 ± 3.04	5.47 ± 4.48	2.63 ± 3.60	1.58 ± 4.75	6.45 ± 4.55	4.20 ± 5.93	5.69 ± 4.68	3.56 ± 5.00
PO3	3.92 ± 3.66	4.45 ± 3.62	7.25 ± 4.02	7.73 ± 5.45	7.46 ± 3.60	6.88 ± 5.45	3.23 ± 3.75	2.15 ± 5.15	7.54 ± 4.71	5.55 ± 5.83	6.10 ± 5.20	4.48 ± 5.44
POz	3.30 ± 3.92	3.85 ± 3.72	7.83 ± 4.13	8.38 ± 5.52	8.44 ± 3.31	7.97 ± 5.43	2.85 ± 4.00	1.64 ± 5.14	8.64 ± 4.57	6.34 ± 5.88	7.32 ± 5.30	5.49 ± 5.36
PO4	4.43 ± 3.49	4.92 ± 3.52	7.29 ± 4.18	7.81 ± 5.19	7.13 ± 3.13	6.44 ± 4.79	3.77 ± 3.87	2.56 ± 5.08	8.00 ± 4.25	5.59 ± 5.95	6.36 ± 4.84	4.26 ± 5.18
O1	4.70 ± 4.57	5.18 ± 4.90	7.07 ± 4.15	7.47 ± 5.74	6.39 ± 3.97	5.89 ± 5.87	4.03 ± 4.06	3.03 ± 5.40	7.02 ± 4.95	5.18 ± 5.83	4.99 ± 5.31	3.42 ± 5.56
Oz	3.61 ± 4.37	4.15 ± 4.58	6.85 ± 4.32	7.30 ± 5.68	6.54 ± 3.94	5.97 ± 5.65	3.22 ± 4.23	2.00 ± 5.27	7.89 ± 4.90	5.76 ± 5.90	5.94 ± 5.20	4.18 ± 5.42
O2	4.83 ± 3.94	5.32 ± 4.40	6.65 ± 4.48	7.04 ± 5.45	5.64 ± 3.44	5.06 ± 4.96	4.14 ± 4.35	2.88 ± 5.14	7.58 ± 4.64	5.25 ± 6.06	5.47 ± 4.83	3.48 ± 5.34

*BD, binge drinking; CG, Control group; CE, correct encoding; IE, incorrect encoding*.

With regard to the Dm effect, the analysis performed in the 200–350 ms interval did not reveal either a main effect or an interaction between Group and Condition factors. In the 350–500 ms latency window, the analysis revealed a significant difference between Regions [*F*(1,48) = 56.37, *p* < 0.001, $\eta_{p}^{2}=0.540$], with larger amplitudes at the parieto-occipital region. There was also a significant Condition by Group interaction [*F*(1,48) = 5.01, *p* = 0.030, $\eta_{p}^{2}=0.094$]; *post hoc* analysis indicated that differences between conditions (larger amplitude for CE than IE, i.e., Dm effect) were only significant in the Control group (*p* = 0.012). Analysis of the 500–650 ms interval revealed a significant main effect of Condition (Dm effect) [*F*(1,48) = 4.83, *p* = 0.033, $\eta_{p}^{2}=0.091$] and Region [*F*(1,48) = 10.14, *p* = 0.003, $\eta_{p}^{2}=0.174$] (larger amplitude at the parieto-occipital region). Although no significant Condition by Group interaction was detected, *post hoc* analysis revealed that differences between conditions were only significant in the Control group (*p* = 0.028).

Statistical analysis of the mean amplitudes in the N170 latency range did not reveal any significant main effects or interactions of the Group or Condition factors. Regarding the VPP component, the analysis revealed significant differences between groups [*F*(1,48) = 4.56, *p* = 0.038], with larger amplitudes in the BD (3.08 µV) than in the Control group (1.64 µV), there were also a main effects of the Electrode factor [*F*(2,96) = 46.89, *p* < 0.001, ε = 0.89, $\eta_{p}^{2}=0.494$] (larger amplitudes at the midline location) and Electrode by Group interaction [*F*(2,96) = 3.46, *p* = 0.036, $\eta_{p}^{2}=0.067$], *post hoc* analysis indicated significant differences between groups at C3 (*p* = 0.015) and Cz (*p* = 0.031) electrodes, with larger amplitudes in BD than Control group.

## Discussion

In the present study, ERPs were used to examine the effects of alcohol BD on declarative memory encoding among undergraduate students, using a subsequent memory paradigm with a visual face–name association memory task.

The results revealed that, despite the absence of behavioral differences between the groups (percentage of associations remembered), the Control and BD groups showed electrophysiological differences during memory encoding. The Control group displayed the classic Dm effect at the 350–500 ms latency window, with larger amplitudes for subsequently remembered face–name pairs than for those that were subsequently forgotten, whereas the BD group did not show this effect, and displayed similar neural activity for successful and unsuccessful encoding. The Dm appeared in the global sample at the 500–650 interval; however, *post hoc* analyses showed that it was only significant in the Control group.

The literature about Dm effect has described significant differences in ERPs between conditions (remembered and unremembered stimuli) during the encoding of verbal and non-verbal material in young healthy people (37, 44, 45). In the present study, significant differences were observed in the Control group, whereas the BD group showed a lack of electrophysiological differences between successful and unsuccessful memory encoding. The absence of differences in neural activity would indicate anomalous processing during this memory stage in young BD subjects that seems to mask the expected Dm effect.

Studies focusing on alcoholic patients have suggested that face–name association learning is impaired in this population (46, 47). Pitel et al. (48) used magnetic resonance imaging with a face–name association task to assess different memory processes, such as face–name memory encoding with different levels of processing (i.e., shallow and deep encoding), showing poorer associative and single-item recognition in alcoholics than in controls.

Regarding BD, neuropsychological (14, 49–51), and fMRI studies (15, 16, 52) have reported impairments in declarative memory among BDs. However, to our knowledge, no previous studies have used ERPs to assess this type of memory in young BDs.

Two previous studies used fMRI to evaluate neural activity during declarative memory in BD subjects. Schweinsburg et al. (15, 16) reported that BDs exhibited increased BOLD activity in frontal and parietal regions and decreased activity in frontal and occipital cortex during memory encoding of new words; however, they did not differentiate items according to subsequent recall. Dager et al. (52) used the subsequent memory paradigm to assess the Dm effect during encoding of visual abstract stimuli. They found that heavy drinkers displayed increased BOLD activity during successful encoding in frontal, posterior parietal, and medial temporal regions, together with less left inferior frontal activation and greater precuneus deactivation during incorrect encoding. Dager et al. (52) suggested that heavy drinkers could show compensatory hyperactivation during correct encoding and greater deactivation of default mode regions during incorrect encoding, which would mean that this population would use different encoding strategies. These results are not in line with our ERP study. However, it should be noted that there were technical and experimental differences between both studies, as they analyzed BOLD activity and they used different stimuli. Moreover, in the study of Dager et al. (52), the sample characteristics were also different, as these authors did not exclude subjects with alcohol use disorder (39.1% of their Heavy Drinkers Group met criteria for this disorder). The results of the two studies are not, therefore, directly comparable.

Previous ERP and fMRI studies have also found neural hyperactivation associated with BD during different cognitive processes, such as working memory (19, 53, 54), inhibitory processes (20, 24, 25), decision-making (17, 55), or reactivity to alcohol-related cues (27, 28). These authors have suggested that the increased activity may be related to the recruitment of additional resources to compensate for underlying neurocognitive deficits in BD. On the contrary, a few other studies have reported smaller amplitudes of ERP components related to perceptual and attentional processes (56) and working memory (23). Accordingly, these authors have suggested that BDs show some neurocognitive anomalies that have been found to be similar in alcoholics, and they have proposed that the maintenance of a BD pattern could be considered a first step toward the development of alcoholism.

In the present study, it is not possible to relate the absence of the Dm effect in the BD group with neural hyper- or hypoactivation, because differences between groups were not significant for the CE or the IE ERP amplitudes; however, they point out to an anomalous activity in regions involved in memory encoding that prevents the emergence of the Dm effect observed in normal population. Further studies are necessary to replicate these results and to clarify whether the absence of Dm is due to abnormal unspecific hyperactivation when encoding fails or to decreased activity when it is successful.

Regarding the inconsistency between behavioral and neural results, most ERP and fMRI studies on BD have found anomalies in neural activity with no behavioral differences between groups (15, 16, 18, 19, 22–24, 26, 27, 53, 54, 56, 57). It has been argued that college BDs who did not develop alcohol dependency manifest subtle deficits that go unnoticed at the behavioral level, but are detected by ERP or neuroimaging techniques. It is possible that subjects who maintain the BD pattern of consumption for a long time may begin to show similar behavioral impairments to those described in patients with alcohol use disorder following after several years of BD.

Although this study focused on components associated with memory encoding, the N170 and VPP were assessed because they are specifically related to perceptual processing of faces (58, 59). Moreover, it should be noted that very few studies on BD have reported anomalies in perceptual ERP components (26, 27, 56). Regard face perception processes, only Maurage et al. (56), in an oddball task using faces as stimuli, reported lower N170 amplitude in high BDs in comparison with light BDs, daily drinkers, and controls. The VPP component was not assessed in that work, and the reported N170 effect was not replicated in the present study. Our results revealed significant differences between groups in the VPP component, with larger amplitudes in BD than Control group. However, in this task each stimulus consisted of a face with a name written below, and face–related and name-related visual ERP components may, therefore, have overlapped. In this sense, these results should be interpreted with caution because we cannot be sure whether the central positive component reflects only face perceptual processes.

In summary, the results of the present study indicate the presence of electrophysiological differences between young college student BDs and controls during the memory encoding process in a visual face–name associative memory task, with an absence of the Dm effect in the BDs. Although the neural significance of these results is not clear, it points, as neuropsychological and fMRI previous evidence, that encoding on declarative memory could be affected by BD in young population. Further studies with larger samples are required to replicate these findings and to further inquiry in its meaning.

## Ethics Statement

The protocol was approved by the Bioethics Committee of the Universidade de Santiago de Compostela. All subjects gave written informed consent in accordance with the Declaration of Helsinki.

## Author Contributions

FC and SRH designed the general study, including sample selection criteria. PP-A implemented the task and wrote a preliminary draft. AC and EL-C collected the data. PP-A, RF-A, and SRH analyzed the data. FC, PP-A, RF-A, and SRH interpreted the data. RF-A wrote the manuscript. All authors have critically revised the manuscript for intellectual content. The final version was approved for publication by all authors.

## Conflict of Interest Statement

The authors declare that the research was conducted in the absence of any commercial or financial relationships that could be construed as a potential conflict of interest.
